# Epidemiological characteristics of 1,806 patients with traumatic spinal cord injury: A retrospective study

**DOI:** 10.3389/fsurg.2022.988853

**Published:** 2023-01-06

**Authors:** Zhihua Wang, Wu Zhou, Meihua Li

**Affiliations:** Department of Neurosurgery, The First Affiliated Hospital of Nanchang University, Nanchang, China

**Keywords:** traumatic spinal cord injury, cervical fracture/dislocation, cervical disc herniation/bulging, prevention, epidemiology

## Abstract

**Background:**

Traumatic spinal cord injury (TSCI) is a type of highly disabling central nervous system trauma. In this study, we investigated the epidemiological characteristics of 1,806 TSCI patients and compared the characteristics of patients with traumatic cervical spinal cord injury (TCSCI) caused by cervical fracture/dislocation and disc herniation/bulging.

**Methods:**

We retrospectively reviewed the hospital records of 1,806 TSCI patients. The detailed information included gender, marital status, occupation, age, neurological level of injury, etiology, American Spinal Injury Association (ASIA) grade, combined injuries, complications, treatment, the interval between admission and surgery, intubation/tracheostomy requirement, and the length of hospital stay.

**Results:**

Cervical spinal cord injury (CSCI) was the most common injury. Compared to non-CSCI cases, patients with TCSCI were older, and more likely to suffer from tetraplegia and require intubation/tracheostomy, but had fewer other injuries or complications and a shorter length of hospital stay. Compared to patients with cervical fracture/dislocation, those with TCSCI caused by disc herniation/bulging were older and more likely to suffer from paraplegia, but required intubation/tracheostomy less frequently, exhibited fewer other injuries and complications, and required shorter hospitalization.

**Conclusions:**

Men, married individuals, manual laborers, and individuals aged 31–75 years had the highest risk of TSCI. Patients with TCSCI tended to have a shorter length of hospital stay than patients with non-CSCI. More attention should be paid to the other injuries and complications of non-CSCI patients, which may increase the length of hospital stay and delay rehabilitation. Compared to patients with cervical disc herniation, the patients with fracture/dislocation tended to be younger, but prognosis was severely compromised by tetraplegia, a greater need for intubation/tracheostomy, additional injuries, and complications.

## Introduction

Central nervous system trauma can cause varying degrees of sensory and motor dysfunction, which seriously affect quality of life and increase the economic burden on the family and society. Traumatic brain injury is common but can occasionally be prevented by wearing a safety helmet to reduce injuries. However, traumatic spinal cord injury (TSCI) sometimes occurs in the absence of effective preventive measures. The life expectancy of spinal cord injury (SCI) patients in the USA has not improved in the past three decades; the overall age-standardized mortality rate from 2010 to 2017 was threefold greater for individuals with SCI than for members of the general population (1). Compared with the 2008 TSCI population profile, Americans living with TSCI during 2015–2019 (mean years since injury: 18 years, 79.4% men, and 62.5% Caucasian) were older (51.6 vs. 45.0 years) and had a higher percentage of C1–C4 (21.9% vs. 17.0%) and American Spinal Injury Association (ASIA) D injuries (31.5% vs. 26.0%) than did individuals in other group (2). In Korea, the mean age (standard deviation) at the time of injury increased from 32.4 (12.4) years in the 1990s to 47.1 (16.2) years in the 2010s. Land transport and falls were the most common causes of TSCI. Tetraplegia was more common than paraplegia; incomplete tetraplegia was highest in the 2010s (3).

TSCI is often caused by falls and traffic accidents, and can induce severe and irreversible dysfunction of both the motor and sensory systems, as well as tetraplegia or paraplegia, and an inability to live unassisted (4, 5). Thus, TSCI impose a major burden on individuals, families, and society, because of the high costs of treatment and rehabilitation and lost productivity (6, 7). Traumatic cervical spinal cord injury (TCSCI) can cause tetraplegia and death. In the acute stage of TCSCI, 84% of patients with C1–4 injuries and 60% with C5–8 injuries experience respiratory complications (8). Timely intubation and tracheostomy are essential (9, 10).

A traumatic cervical spinal fracture (TCSF) is typically caused by severe violence; if this is combined with a dislocation, the risk of CSCI is greatly increased. The mean annual incidence of TCSF is 65 cases per 100,000 hospital admissions; risk factors include an age of 31–45 years, male sex, fall from a height, and traumatic [C5, C6] vertebral fractures (11). The intervertebral discs separate the vertebral bodies and evenly spread the loads among them. These discs degenerate with age and become more susceptible to injury (12). TCSF/dislocation has received a great deal of attention worldwide (13–15). However, cervical disc herniation and bulging have not been well-studied. The posterior ligamentous complex includes the intervertebral disc, ligamentum flavum, and interspinous and nuchal ligaments; this complex plays a critical role in cervical spine stability (16, 17). This retrospective and descriptive study examined the epidemiological data of 1,806 TSCI patients who were admitted from 1 January 2012 to 31 December 2020. We also retrospectively analyzed the epidemiology of TCSCI and non-CSCI, and compared the clinical characteristics of patients with TCSCI caused by cervical fracture/dislocation and disc herniation/bulging.

## Patients and methods

Two researchers separately reviewed the data of all hospitalized patients diagnosed with SCI. Detailed information were recorded, including gender, marital status, occupation, age, neurological level of injury, etiology, American Spinal Injury Association (ASIA) grade, combined injuries, complications, treatment, the interval between admission and surgery, intubation/tracheostomy requirement, and the length of hospital stay. The inclusion criteria were spinal cord or cauda equina injury, confirmed by clinical symptoms and an imaging examination or surgery; injury resulting from trauma; and reasonable and detailed patient records. The exclusion criteria were: non-traumatic spinal cord or cauda equina injury; treatment in departments other than orthopedics or neurosurgery; and unreasonable or incomplete patient records.

In this study, the TCSCI group included only patients with simple cervical injuries. The non-CSCI group included patients with injuries in the thoracic, lumbar, cervical and thoracic; thoracic and lumbar; cervical and lumbar; and cervical, thoracic and lumbar regions. The fracture/dislocation group included patients with at least one cervical vertebral fracture/dislocation other than disc herniation/bulging. Patients in the disc herniation/bulging group exhibited herniation/bulging of at least one cervical disc without any fracture/dislocation. SPSS software version 25.0 and GraphPad Prism software version 8.0 were used for the data analysis. The data are presented as numbers, percentages and medians with interquartile range (IQR). The Mann-Whitney U test and chi-squared (*χ*^2^) test were used to compare the two groups; the level of statistical significance was set to *p* < 0.05.

## Results

**Figure 1:**
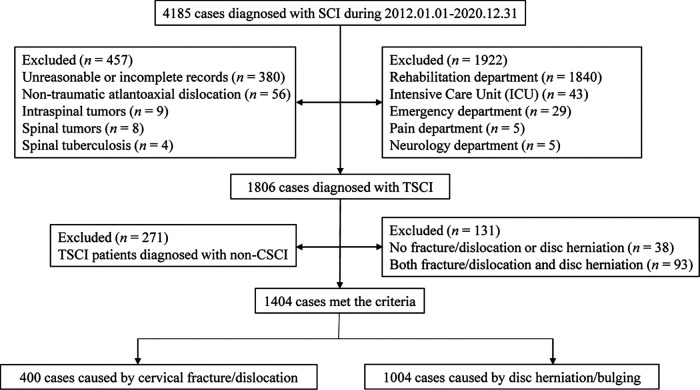
Flow chart of patient selection.

The flow chart for patient selection is displayed in Figure 1. In total, 4,185 patients were diagnosed with SCI; of these, 1,806 were diagnosed with TSCI, 2,379 were excluded for some reasons. 1,922 cases were excluded because they involved treatment in departments other than orthopedics or neurosurgery. 380 were excluded because of unreasonable or incomplete medical records. 77 cases were excluded because of non-traumatic factors.

### Gender and age

**Figure 2:**
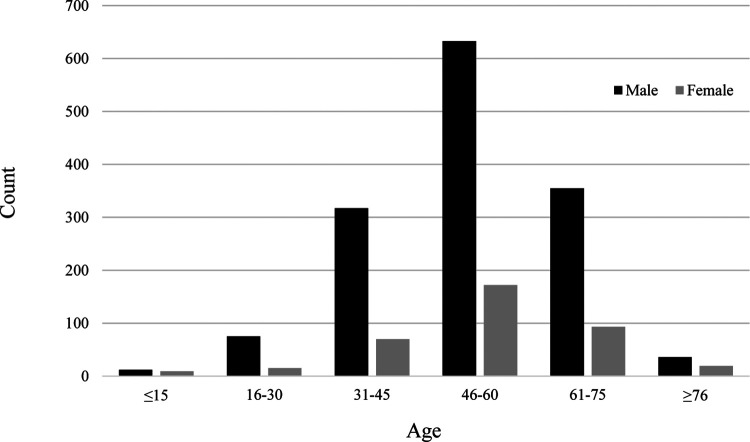
High-risk age group of different genders.

The ratio of men to women was 3.8:1. The patients were divided into six age groups: ≤ 15, 16–30, 31–45, 46–60, 61–75, and ≥ 76 years. The median age (IQR) of the 1,806 patients was 53 (IQR = 45–61) years [men: 53 (IQR = 44–61), women: 53 years (IQR = 46–62)]. The three age groups with the largest numbers of patients were 46–60, 61–75, and 31–45 years (Table 1). Patients in these groups constituted 90.9% of all included patients. The high-risk age group was similar for men and women (Figure 2). The 46–60 years age group was the largest, followed by the 61–75 years and 31–45 years age groups.

**Table 1:** Characteristics of 1,806 TSCI patients.

Variables		No.	Pct. (%)
Gender	Male	1428	79.1
	Female	378	20.9
Marital status	Married	1661	92.0
	Unmarried	89	4.9
	Others	56	3.1
Occupation	Peasant	1488	82.4
	Worker	119	6.6
	Civil servant	5	0.3
	Student	85	4.7
	Others	109	6.0
Age	≤ 15	21	1.1
	16–30	90	5.0
	31–45	386	21.4
	46–60	808	44.7
	61–75	447	24.8
	≥ 76	54	3.0
Level of injury	Cervical	1535	85.0
	Thoracic	122	6.8
	Lumbar	83	4.6
	Cervival + Thoracic	37	2.0
	Thoracic + Lumbar	19	1.1
	Cervival + Lumbar	8	0.4
	Cervical + Thoracic + Lumbar	2	0.1
Etiology	Fall	924	51.2
	Traffic accident	508	28.1
	Struck by object	82	4.6
	Others	292	16.1
ASIA grade	A	357	19.8
	B	113	6.3
	C	631	34.9
	D	649	35.9
	E	56	3.1
Combined injury	Yes	787	43.6
	No	1019	56.4
Complication	Yes	385	21.3
	No	1421	78.7
Treatment	Conservation	303	16.8
	Surgery	1503	83.2

### Marital Status, occupation, and etiology

**Figure 3:**
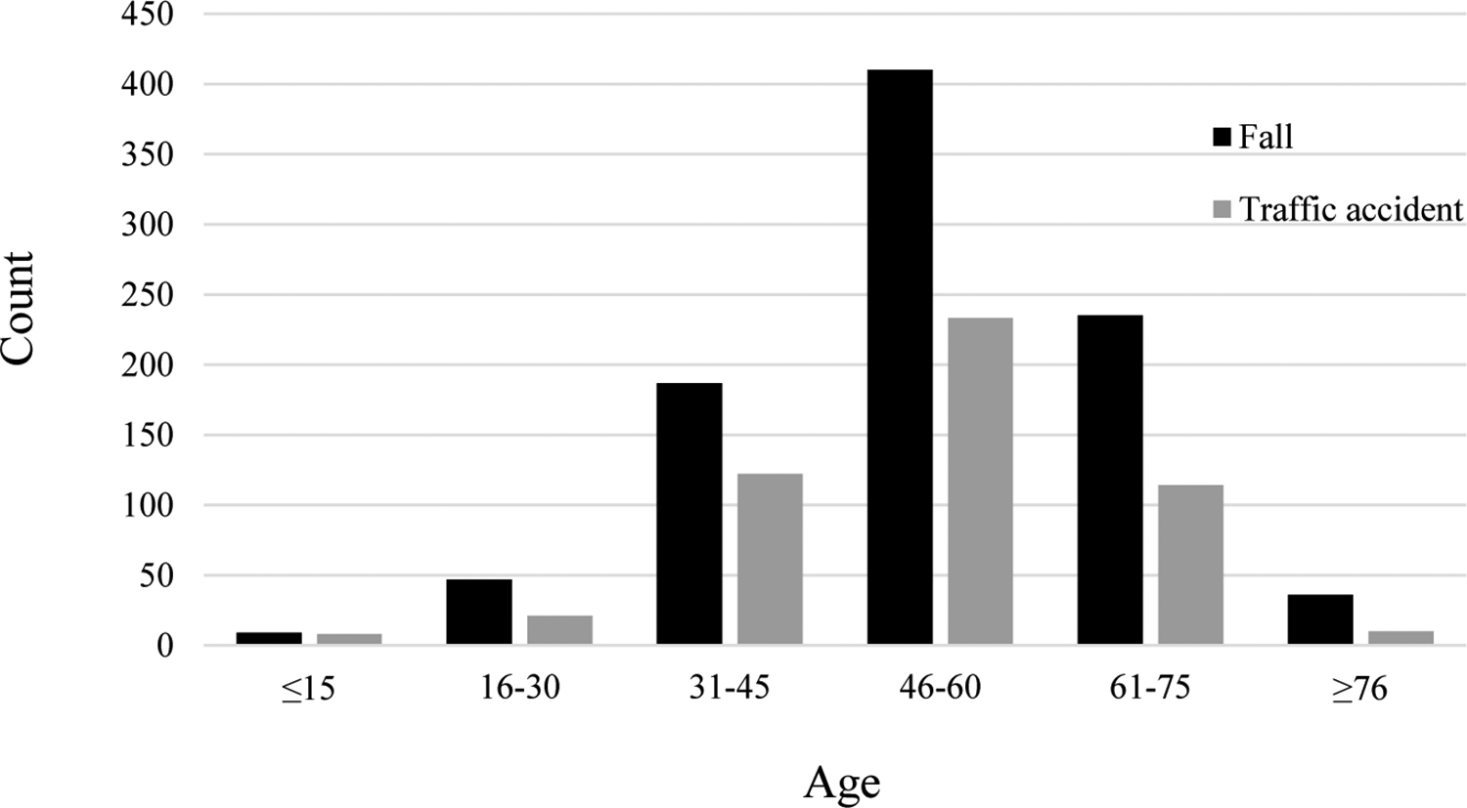
High-risk age group of the top two causes.

As shown in Table 1, 92.0% of patients were married. Manual laborers (peasant and worker, 89.0%) were the main group of TSCI patients. Falls (51.2%) and traffic accidents (28.1%) were the two most common causes of TSCI. Patients with impact-related injuries included 72 who were struck by objects falling from a high altitude, 6 who received lateral impacts because of operating errors during work, and 4 who received cattle impacts. Other patients experienced knife wounds (*n* = 5), fighting injuries (*n* = 7), and unclassified trauma (*n* = 280). The high-risk age group experienced similar rates of the two most common causes (Figure 3). The data had a unimodal distribution and both peaks were in the 46–60 years age group.

### Level of injury and ASIA grade

The SCI segment was determined on the basis of clinical symptoms, computed tomography and magnetic resonance imaging findings, or postoperative records. The cervical spinal cord was more often injured than the thoracic or lumbar segments, with a total operation rate of 73.4% (1227/1740). Few cases were encountered involving combined (i.e., simultaneous) injuries of two or three segments, but their operation rate was higher (93.9%; 62/66). ASIA grades D and C constituted 70.8% of the total cases (Table 1).

### Combined injuries, complications and treatment

Approximately 44% of the TSCI patients had at least one combined injury upon admission to the hospital (Table 1). As shown in Table 2, the percentage of trunk injuries was higher than the percentages of traumatic brain injury or limb injuries. Pulmonary contusion, hydropneumothorax, and rib fracture were the three most common trunk injuries. The most common traumatic brain injury was a cerebral contusion and laceration, while tibiofibular fracture was the most common limb injury. In total, 21.3% of patients had at least one complication during their hospitalization (Table 1). The percentage of complications is shown in Table 3. The three most common complications were pulmonary infection, pleural effusion, and respiratory failure. Among the 1,806 TSCI patients treated in our hospital, the operation rate was 83.2% (Table 1). In total, 303 patients were not treated because they discontinued treatment (*n* = 156), were transferred to a specialized rehabilitation department or hospital (*n* = 142), or were transferred to another large hospital (*n* = 5).

**Table 2:** Combined injuries of 787 TSCI patients.

Combined injuries	No.	Pct. (%)
Trunk injuries (Chest, abdomen and pelvis)	Pulmonary contusion	302	38.4
	Hydropneumothorax	268	34.1
	Rib fracture	129	16.4
Traumatic brain injuries	Cerebral contusion and laceration	274	34.8
	Traumatic subarachnoid hemorrhage	203	25.8
	Skull fracture	115	14.6
Limb injuries	Tibiofibula fracture	84	10.7
	Ulna radial fracture	57	7.2
	Ankle injury	21	2.7

**Table 3:** Clinical complications of 385 TSCI patients.

Complications	No.	Pct. (%)
1	Pulmonary infection	306	79.5
2	Pleural effusion	123	31.9
3	Respiratory failure	100	26.0
4	Urinary tract infection	31	8.1
5	Bedsore	16	4.2
6	Septic shock	14	3.6
7	Deep venous thrombosis	12	3.1
8	Death	9	2.3
9	Intracranial infection	5	1.2
10	Pulmonary embolism	2	0.5
11	Paralytic ileus	2	0.5

### Characteristics of the 1,806 patients with different ASIA grades

As shown in Table 4, the 46–60 years age group had the greatest proportion of different ASIA grades. CSCI was much more frequent than SCI involving other segments. The proportion of patients who underwent surgery within 24 h after admission was low; most patients underwent surgery within 4–7 days after admission. Nearly 90% of patients in each grade underwent surgery within 7 days of admission. The artificial airway management status and the mean in-hospital stay findings were consistent with injury severity. Patients with a worse ASIA grade were more likely to receive tracheostomy or intubation and have a longer mean in-hospital stay.

**Table 4:** Characteristics of 1,806 patients with different ASIA grade.

Variables	No. & Pct. (%)	ASIA grade				
		A	B	C	D	E
Age	≤ 15	6 (1.7)	4 (3.5)	6 (0.9)	4 (0.6)	1 (1.8)
	16–30	30 (8.4)	5 (4.4)	26 (4.1)	26 (4.0)	3 (5.4)
	31–45	79 (22.1)	17 (15.0)	134 (21.2)	140 (21.6)	16 (28.6)
	46–60	156 (43.7)	48 (42.5)	266 (42.2)	309 (47.6)	29 (51.8)
	61–75	81 (22.7)	37 (32.7)	171 (27.1)	152 (23.4)	6 (10.7)
	≥ 76	5 (1.4)	2 (1.9)	28 (4.4)	18 (2.8)	1 (1.7)
	Total	357 (100)	113 (100)	631 (100)	649 (100)	56 (100)
Level of injury	Cervical	257 (72.0)	90 (79.6)	560 (88.7)	591 (91.1)	37 (66.1)
	Thoracic	63 (17.6)	16 (14.2)	22 (3.5)	15 (2.2)	6 (10.7)
	Lumbar	14 (3.9)	5 (4.4)	28 (4.4)	30 (4.5)	6 (10.7)
	Cervival + Thoracic	15 (4.2)	1 (0.9)	12 (1.9)	7 (1.1)	2 (3.6)
	Cervival + Lumbar	2 (0.6)	0	3 (0.5)	3 (0.5)	0
	Thoracic + Lumbar	5 (1.4)	1 (0.9)	6 (1.0)	2 (0.4)	5 (8.9)
	Cervical + Thoracic + Lumbar	1 (0.3)	0	0	1 (0.2)	0
	Total	357 (100)	113 (100)	631 (100)	649 (100)	56 (100)
Time from admission to surgery	≤ 24 h	91 (29.6)	14 (14.4)	65 (11.8)	34 (6.7)	5 (13.5)
	2day-3day	96 (31.3)	29 (29.9)	194 (35.2)	191 (37.4)	10 (27.0)
	4day-7day	88 (28.7)	45 (46.4)	237 (43.0)	228 (44.6)	18 (48.6)
	>7day	32 (10.4)	9 (9.3)	55 (10.0)	58 (11.3)	4 (10.9)
	Total	307 (100)	97 (100)	551 (100)	511 (100)	37 (100)
Intubation or tracheostomy	Yes	90 (25.2)	13 (11.5)	45 (7.1)	11 (1.7)	0
	No	267 (74.8)	100 (88.5)	586 (92.9)	638 (98.3)	56 (100)
	Total	357 (100)	113 (100)	631 (100)	649 (100)	56 (100)
Mean in-hospiyal day		23.9	19.2	17.8	13.8	13.7

### Characteristics of TSCI and TCSCI

**Figure 4:**
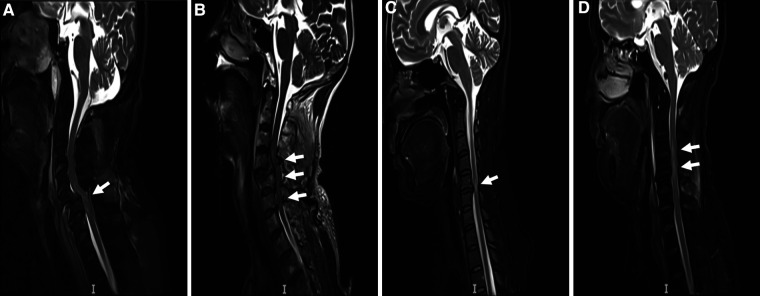
Two typical MRI scans of TCSCI patients. MRI scans of TCSCI patients with fracture/dislocation and disc herniation/bulging. (**A**) The C5 vertebral body slipped forward to the III° position; the spinal canal was narrowed and the cervical spinal cord was severely compressed. (**B**) The C4 vertebral body slipped forward to the II° position and a TCSCI is evident at the C4 -6 level. (**C**) The C5/6 intervertebral disc herniated backward and a TCSCI is apparent at the C5/6 level, especially C5. (**D**) The C3/4, C4/5, C5/6, and C6/7 intervertebral discs herniated posteriorly and a TCSCI is apparent at the C3/C4 level.

**Figure 5:**
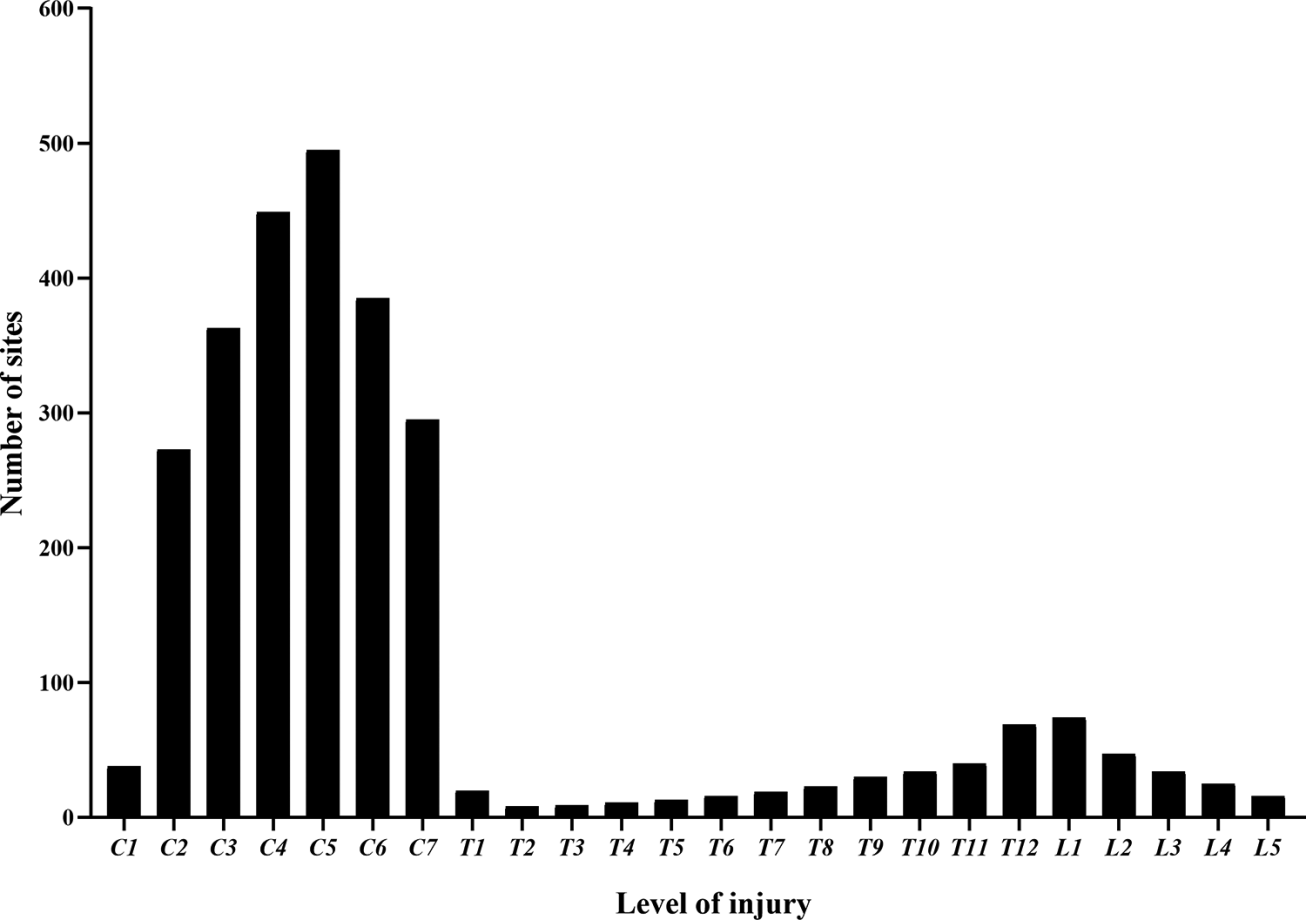
Distributions of sites of injury (C1–L5).

**Figure 6:**
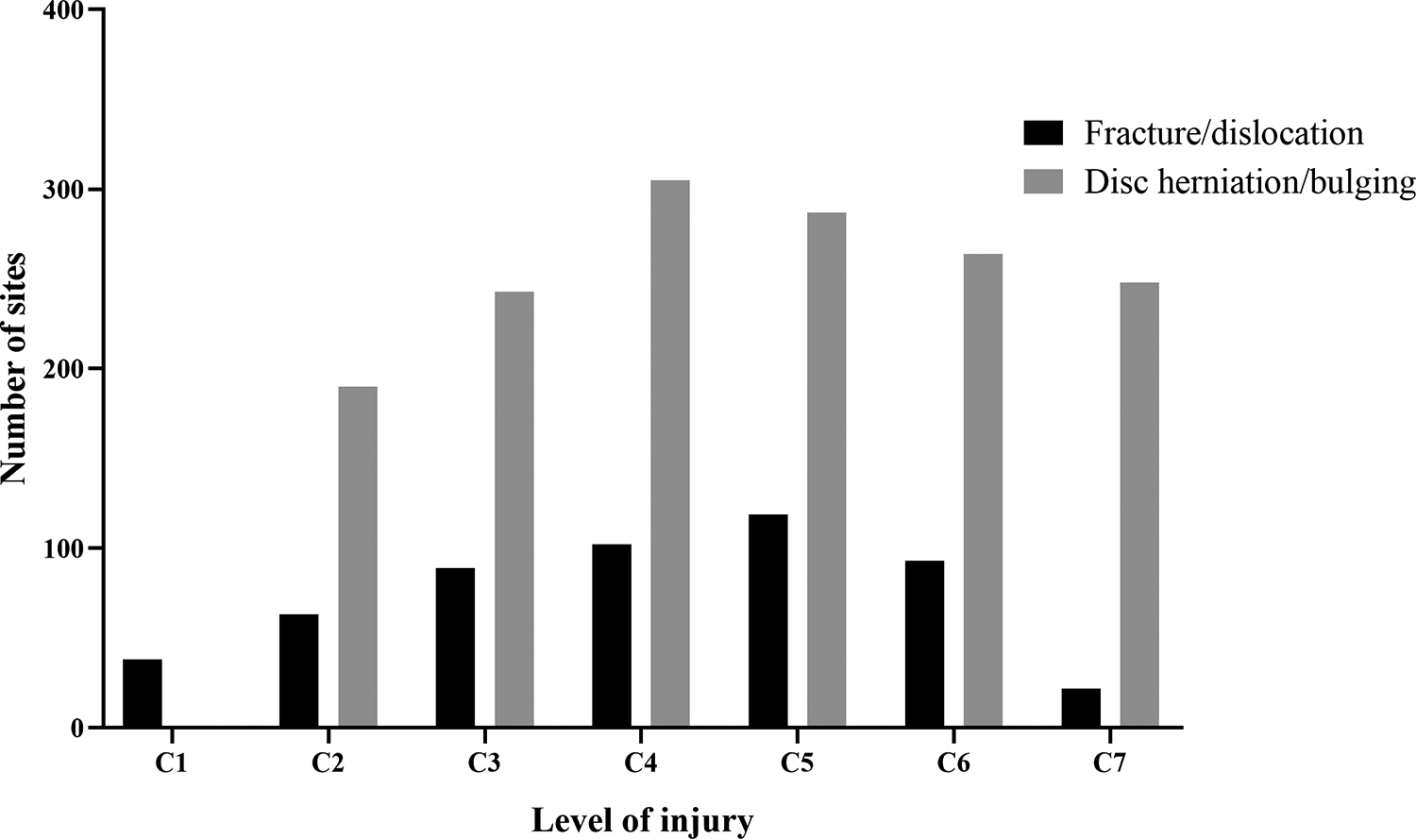
Distributions of all sites of injury (C1–C7) caused by fracture/dislocation and disc herniation/bulging.

Figure 1 shows that 1,806 patients were diagnosed with TSCI, some of whom had multi-segment or multi-site injuries (Figure 4). All injury sites were detected *via* computed tomography (CT) or magnetic resonance imaging (MRI), or postoperatively; and the most common injuries were CSCI (Figure 5). There were 1,535 patients in the TCSCI group and 271 in the non-CSCI group. We focused on 400 TCSCI patients (526 sites of injury) who suffered cervical fracture/dislocation and 1,004 patients (1,537 sites of injury) with cervical disc herniation/bulging (Figure 6).

### Characteristics of the 1,806 patients with TCSCI and non-CSCI

Table 5 compares the medical characteristics of the 1,535 TCSCI patients to those of the 271 non-CSCI patients. In the TCSCI group, the male: female ratio was 4.0:1, the age group with the highest risk was the 46–60 years group (followed by the 61–75 and 31–45 years groups), the tetraplegia rate was 90.6% and most patients were of ASIA grade D or C (in that order). In the non-CSCI group, the male: female ratio was 2.7:1, the age group with the highest risk was the 46–60 years group (followed by the 31–45 and 61–75 years groups), the paraplegia rate was 80.1% and most patients were of ASIA grade A or C (in that order). Patients with TCSCI (median age, 54 years; IQR = 46–62 years) tended to be older than those with non-CSCI (median age, 48 years; IQR = 37–57 years) (*p* < 0.001). Significant differences between the TCSCI and non-CSCI patients were seen for gender, age, neurological injury level, ASIA grade, interval between admission and surgery, other injuries, complications, and the length of hospital stay, but not in treatment or the intubation/tracheostomy requirement.

**Table 5:** Characteristics of 1,806 patients resulted from TCSCI and Non-CSCI.

Variables	TCSCI group *n* (%)	Non-CSCI *n* (%)	*p* value
Gender	Male	1,231 (80.2)	197 (72.7)	*χ*^2^ = 7.832, *P* = 0.005
	Female	304 (19.8)	74 (27.3)	
Age	≤ 15	11 (0.7)	10 (3.7)	χ^2^ = 67.125, *P* < 0.001^#^
	16–30	55 (3.6)	35 (12.9)	
	31–45	312 (20.3)	74 (27.3)	
	46–60	703 (45.8)	105 (35.1)	
	61–75	402 (26.2)	45 (16.6)	
	≥ 76	52 (3.4)	2 (4.4)	
Neurological level of injury	Tetraplegia	1,390 (90.6)	54 (19.9)	χ^2^ = 716.915, *P* < 0.001
	Paraplegia	145 (9.4)	217 (80.1)	
ASIA grade	A	256 (16.7)	101 (37.3)	χ^2^ = 93.978, *P* < 0.001
	B	90 (5.9)	23 (8.5)	
	C	561 (36.5)	70 (25.8)	
	D	591 (38.5)	58 (21.4)	
	E	37 (2.4)	19 (7.0)	
Time from admission to surgery	≤ 24 h	155 (12.1)	54 (23.9)	χ^2^ = 24.449, *P* < 0.001
	2day-3day	442 (34.6)	78 (34.5)	
	4day-7day	539 (42.2)	77 (34.1)	
	>7day	141 (11.1)	17 (7.5)	
Combined injury	Yes	600 (39.1)	187 (69.0)	χ^2^ = 83.839, *P* < 0.001
	No	935 (60.9)	84 (31.0)	
Complication	Yes	310 (20.2)	75 (27.7)	χ^2^ = 7.683, *p* = 0.006
	No	1,225 (79.8)	196 (72.3)	
Treatment	Surgery	1,277 (83.2)	226 (83.4)	χ^2^ = 0.007, *p* = 0.934
	Conservation	258 (16.8)	45 (16.6)	
Intubation or tracheostomy	Yes	140 (9.1)	19 (7.0)	χ^2^ = 1.277, *p* = 0.259
	No	1,395 (90.9)	252 (93.0)	
Length of stay (IQR)		13 (10, 20)	18 (12, 26)	*p* < 0.001*

IQR, interquartile range.

*Mann-Whitney *U* test.

^#^Fisher's exact test.

### Characteristics of the 1,404 patients with cervical fracture/dislocation and disc herniation/bulging

Table 6 summarizes the medical characteristics of the 1,404 TCSCI patients with cervical fracture/dislocation and disc herniation/bulging. In the cervical fracture/dislocation group, the male: female ratio was higher than in the other group (5.5:1 vs. 3.5:1). Patients with cervical disc herniation/bulging were older (median age, 54 years; IQR = 47–62.75 years) compared to the other patients (median age, 51 years; IQR = 42.25–61 years) (*p* < 0.001). In the fracture/dislocation group, the rate of tetraplegia was higher than in the other group (93.5% vs. 89.2%) and most patients were of ASIA grade C. In the disc herniation/bulging group, the rate of paraplegia was higher than in the other group (10.8% vs. 6.5%) and most patients were of ASIA grade D. With the exception of treatment, there were significant group differences in all other parameters.

**Table 6:** Characteristics of 1,404 patients caused by fracture/dislocation and disc herniation/bulging.

Variables	Fracture/dislocation group *n* (%)	Disc herniation/bulging group *n* (%)	*p* value
Gender	Male	338 (84.5)	779 (77.6)	χ^2^ = 8.399, *p* = 0.004
	Female	62 (15.5)	225 (22.4)	
Age	≤ 15	7 (1.8)	3 (0.3)	χ^2^ = 66.414, *P* < 0.001^#^
	16–30	37 (9.3)	13 (1.3)	
	31–45	99 (24.7)	187 (18.6)	
	46–60	152 (38.0)	489 (48.7)	
	61–75	93 (23.2)	276 (27.5)	
	≥ 76	12 (3.0)	36 (3.6)	
Neurological level of injury	Tetraplegia	374 (93.5)	896 (89.2)	χ^2^ = 6.004, *p* = 0.014
	Paraplegia	26 (6.5)	108 (10.8)	
ASIA grade	A	116 (29.0)	110 (11.0)	χ^2^ = 76.866, *p* < 0.001
	B	15 (3.8)	56 (5.6)	
	C	142 (35.5)	380 (37.8)	
	D	114 (28.5)	434 (43.2)	
	E	13 (3.2)	24 (2.4)	
Time from admission to surgery	≤ 24 h	58 (17.9)	81 (9.6)	χ^2^ = 18.430, *p* < 0.001
	2day-3day	99 (30.6)	298 (35.4)	
	4day-7day	124 (38.3)	373 (44.3)	
	>7day	43 (13.2)	90 (10.7)	
Combined injury	Yes	173 (43.3)	354 (35.3)	χ^2^ = 7.790, *p* = 0.005
	No	227 (56.7)	650 (64.7)	
Complication	Yes	122 (30.5)	164 (16.3)	χ^2^ = 35.384, *p* < 0.001
	No	278 (69.5)	840 (83.7)	
Treatment	Surgery	324 (81.0)	842 (83.9)	χ^2^ = 1.667, *p* = 0.197
	Conservation	76 (19.0)	162 (16.1)	
Intubation or tracheostomy	Yes	53 (13.3)	83 (8.3)	χ^2^ = 8.119, *p *= 0.004
	No	347 (86.7)	921 (91.7)	
Length of stay (IQR)		15 (10, 24)	13 (9, 19)	*p* < 0.001*

IQR: interquartile range.

*Mann-Whitney U test

^#^Fisher's exact test

## Discussion

After careful selection, we identified 1,806 cases; the ratio of men to women in this study was 3.8:1, which was similar to the ratios in several previous studies in China (18–22). In other regions, the male: female ratios have been 2.4:1 in Russia, 2.6:1 in Finland, 3.0:1 in Japan, 3.6:1 in Mexico, 6.1:1 in India, and 7.3:1 in Saudi Arabia (23–28). China is the largest developing country in the world. The agricultural population of China has an absolute numerical advantage. Middle-aged and elderly people are always the “backbone of the family”; they engage in manual labor. In this study, married individuals, manual laborers, and individuals aged 31–75 years (especially 46–60 years) had the highest risk of TSCI. A previous comparative study indicated that TSCI caused by low and high falls has distinct epidemiological characteristics (29). In our study, some medical records were marked “fall” without a specific height or marked “traffic accident” without a specific vehicle type; they could not be further stratified into other subtypes. However, the results were consistent with previous findings; fall and traffic accidents were the two most common causes of TSCI (11, 18, 20, 22, 30–32).

CSCI is recognized as the most frequent type of TSCI. In this study, we examined the types and occurrence of combined injuries and complications to provide more clinically useful insights. Although surgical decompression should be performed as soon as possible (with the ideal surgical timing of 8 h post-injury) for both complete and incomplete lesions), many patients in this study underwent surgery within 4–7 days after admission (33–35). Possible reasons for such delay were as follows. First, many patients needed to maintain their physical status and stabilize their condition to reduce the risk of death after hospital admission. Second, imaging examinations and preoperative examinations (i.e., magnetic resonance imaging) could not be performed immediately. Third, specialists explained the need for surgery, but the family members required several days of consideration prior to consent for surgery. Our results also indicate that patients with a worse ASIA grade were more likely to undergo airway management with a longer mean in-hospital stay.

To the best of our knowledge, this is the first study to classify and compare the clinical characteristics of patients with TCSCI caused by fracture/dislocation and disc herniation/bulging. We first compared the clinical features of TCSCI and non-CSCI patients. The treatments and intubation/tracheostomy requirements did not differ between the two groups. A comparison of two subgroups of TCSCI patients indicated that those with fracture/dislocation were more likely to require intubation/tracheostomy. Treatment did not show a group difference, perhaps because all patients underwent careful initial evaluation followed by surgery if necessary.

A study conducted in Chongqing (China) reviewed 643 patients with TCSI; the mean age was 42.5 ± 13.8 years (range: 18–86 years), the male: female ratio was 4.3:1 and the most common site of injury was C5 (22.7% of cases) (11). A study conducted in Maryland (USA) evaluated 1,420 patients with TCSCI; 78.3% were male, with a mean age of 51.5 years. Complete TCSCI were noted in 29.6% of cases, and fracture dislocations were apparent in 44.7% (36). The incidence of traumatic disc herniation was 32% (37). In the present study, we screened more TCSCI patients with cervical disc herniations than fractures [71.5% (1,004/1,404) vs. 28.5% (400/1,404)]. The former patients were older (median age, 54 years; IQR = 47–62.75 years vs. 51 years; IQR = 42.25–61 years), as reflected in the higher proportion of patients aged 46–60 years (48.7% vs. 38.0%), and accounted for 71.5% (1,004/1,404) of all TCSCI cases. In general, the injuries were not as serious as those of patients with traumatic cervical fracture/dislocation. Thus, the rates of tetraplegia and operation within 24 h were lower in the former group, as were the intubation/tracheostomy rates and those of other injuries and complications. This may explain the short hospitalization period. These patients are quickly transferred for rehabilitation. Shorter hospitalization and rapid rehabilitation reduce the incidence of complications. Thus, in daily practice, patients with severe cervical fracture/dislocation require particular attention in terms of active treatment, including of other injuries, and prevention of complications. For patients with TCSCI caused by cervical disc herniation/bulging, early surgery, recovery, and rehabilitation are possible.

Notably, this study did not include data regarding TSCI patients from other affiliated hospitals or large grade A tertiary hospitals in Jiangxi Province. We only examined the epidemiological characteristics of some TSCI patients; thus, these findings are limited to the current status of diagnosis and treatment of TSCI patients in our hospital.

## Data Availability

All data generated or analysed are included in this article and are available from the corresponding author on reasonable request.
